# IRAK-M Deficiency Exacerbates Ischemic Neurovascular Injuries in Experimental Stroke Mice

**DOI:** 10.3389/fncel.2018.00504

**Published:** 2018-12-21

**Authors:** Chenfei Lyu, Yongfang Zhang, Minhua Gu, Yusheng Huang, Guanghui Liu, Chen Wang, Miaodan Li, Shumin Chen, Suyue Pan, Yong Gu

**Affiliations:** ^1^Department of Neurology, Nanfang Hospital, Southern Medical University, Guangzhou, China; ^2^School of Chinese Medicine, Southern Medical University, Guangzhou, China; ^3^Department of Spinal Surgery, Nanfang Hospital, Southern Medical University, Guangzhou, China; ^4^Department of Encephalopathy, Hainan Provincial Hospital of Traditional Chinese Medicine, Haikou, China

**Keywords:** cerebral ischemia, IRAK-M, blood–brain barrier, proinflammatory cytokines, NF-κB

## Abstract

**Background:** Innate immune response to neuronal death is one of the key events of the pathogenesis of ischemic brain injury. Interleukin-1 receptor-associated kinase (IRAK)-M, encoded by gene *Irak3*, negatively regulates toll-like receptor signaling by interacting with the MyD88–IRAK-4–IRAK-1 complex and blocking the phosphorylation and dissociation of IRAK-1. Its function in the ischemic stroke is unknown.

**Objective:** This study aims to investigate whether IRAK-M deficiency could exacerbate neuroinflammation and neurovascular injuries during cerebral ischemia and reperfusion.

**Methods:** Male C57BL/6 mice and *Irak3* knockout mice were subjected to 45 min of middle cerebral artery occlusion and 4 or 24 h of reperfusion. Transcription of *Irak3* gene was evaluated by quantitative real-time PCR (qRT-PCR). Then, infarct volume, neurological score, brain water content, and Evans blue leakage were compared between knock-out and wild-type mice after reperfusion. Through the observation of gross brain specimen after cerebral ischemia, the incidence of hemorrhage transformation was compared between KO and WT mice. To explore underlying signaling pathways involved in IRAK-M deficiency, major proinflammatory cytokines and NF-κB signaling were measured by qRT-PCR and Western blot.

**Results:** The expression of IRAK-M peaked at 1 h after reperfusion, and then gradually decreased within the first 24 h, which was abolished by blocking the expression of hypoxia induced factor 1α. IRAK-M deficiency increased infarct volume, brain edema, the incidence of hemorrhage transformation, and the permeability of blood–brain barrier. In addition, the NF-κB-mediated expressions of proinflammatory cytokines and the activation of microglia in the ipsilateral brain from knock-out mice were much higher than those in wild-type littermates.

**Conclusion:** IRAK-M deletion exacerbates neurovascular damages which are related to the pronounced activation of NF-κB signaling and neuroinflammatory responses during cerebral ischemia-reperfusion in mice. Our study indicates that IRAK-M has neuroprotective effect and has potential to facilitate the development of new pharmaceuticals that reduce neurovascular complications.

## Introduction

Stroke is one of the leading causes of death and disability worldwide. With the increasing aging of population, the stroke incidence rate is projected to increase. Although a complicated sequence of pathophysiological events is involved in the pathogenesis of ischemic stroke, accumulating evidence demonstrates that inflammatory response is one of the key events in the ischemic brain injury and may offer potential therapeutic target for stroke therapy (Lakhan et al., 2009; Iadecola and Anrather, 2011). At the beginning of the focal perfusion defect, shear stress, oxidative stress, and hypoxia initiate both adaptive and innate inflammatory response (Gu et al., 2014). In this process, rapidly activated resident immune cells release multiple cytokines, which are responsible for neuron necrosis and injuries to the supportive structures in the brain (Eltzschig and Eckle, 2011), leading to the brain lesion caused by ischemia.

In the acute phase of cerebral ischemia, the innate immune response is triggered after the recognition of danger-associated molecular patterns by PRRs on macrophage or microglia (Garcia-Bonilla et al., 2014; Liesz et al., 2015). TLRs, a crucial family of PRR, play pivotal roles in inflammation via the activation of NF-κB, type I interferon, and MAPK signaling pathways (Akira and Takeda, 2004; Tang et al., 2007; Eltzschig and Eckle, 2011). Among them, the activation of TLR4 enhances the neuroinflammation and enlarges infarct size in experimental stroke model (Caso et al., 2007; Zhao et al., 2018). MyD88-dependent signaling pathway plays a key role in TLR4-mediated neuroinflammation after cerebral ischemia reperfusion (Gao et al., 2009). After being recruited to the TIR domain of TLR4, MyD88 binds to interleukin receptor associated kinase (IRAK) family, which activates cascades of downstream kinases and contributes to the activation of NF-κB (Deng et al., 2000). Upon TLRs activation, microglial cells release multiple inflammatory factors including TNF-α, IL-1β, iNOS, COX-2, MMPs, and etc. Subsequently, these factors destroy BBB and neurovascular unit.

IRAK-M encoded by *Irak3* negatively regulates TLRs signaling and ameliorates the inflammatory tissue injury (Kobayashi et al., 2002). Upon TLR4 ligation, the recruitment and formation of MyD88-IRAK-4-IRAK-1 complex (also called Myddosome) leads to IRAK1 phosphorylation by IRAK4 and its dissociation from complex. The free phosphorylated IRAK1 then binds to TRAF6 and activates IKK and NF-κB-dependent proinflammatory signaling. IRAK-M interacts with Myddosome to prevent the phosphorylation and dissociation of IRAK-1, interrupts the formation of the IRAK1-TRAF6 complex, and inhibits the activation of NF-κB signaling (Akira and Takeda, 2004; Liu et al., 2008). In addition to the established IRAK1 dependent pathway, IRAK-M is also reported to prevent the NF-κB-inducing kinase (NIK)–IKK α-mediated alternative NF-κB pathway (Su et al., 2009) and TLR7-induced MEKK 3-dependent second wave NF-κB activation (Zhou et al., 2013). IRAK-M not only plays a critical role in immune related diseases including chronic alcoholic liver disease (Zhou et al., 2016), inflammatory bowel diseases (Weersma et al., 2007), and allergic airway inflammation (Nechama et al., 2018), but is also associated with the ischemic tolerance in the model of liver (Izuishi et al., 2006) and myocardial ischemia-reperfusion injury (Chen et al., 2012). Our previous research demonstrated that TLR4/IRAK-M pathway is possibly involved in cerebral ischemic tolerance induced by recombinant high mobility group box 1 preconditioning (Wang et al., 2016). However, up to now, little is known about how to induce IRAK-M and its function in the brain.

*Irak3* expression is induced by several transcription factors. HIF-1α is a master regulator in response to hypoxia and ischemia (Chavez and LaManna, 2002), and is reported to regulate IRAK-M expression in monocytes in response to Lipid A (LPA, a specific TLR4 ligand) (Shalova et al., 2015). In the present study, we found that ischemia-induced HIF-1α is involved in the transcription of *Irak3*. Then we used *Irak3*-null mice for the first time to investigate the role of IRAK-M in cerebral ischemia, and found IRAK-M deficiency increased infarct volume, brain edema, incidence of hemorrhage transformation, BBB permeability, and levels of inflammatory responses.

## Materials and Methods

### Animals

*Irak3* KO mice on C57BL/6 background were obtained from Jackson Laboratories (Bar Harbor, ME, United States, RRID:IMSR_JAX:007016) and WT littermates were generated through heterozygous mating. Mice were housed in a SPF environment with a 12 h light/dark cycle and a temperature of (22 ± 1°C) and were offered free access to food and water. *Irak3* deficient mice developed severe osteoporosis (Li et al., 2005), increased inflammatory responses to bacterial infection (Kobayashi et al., 2002), promoted adverse remodeling and worse systolic dysfunction in myocardial infarction (Chen et al., 2012), and enhanced the development of type 1 diabetes in NOD mice (Tan et al., 2014). DNA of WT and KO mice was extracted from mouse tails with a genomic DNA kit (CoWin Biotech, Beijing, China). To confirm the genotype of mice, extracted DNA was amplified by DNA polymerase (Takara, Kusatsu, Japan) and then separated by agarose gel. Male WT and KO mice (age 7–10 weeks; weight 22–26 g) were used for the experiments. Randomization was performed by a third person unrelated to the study using a randomization table. Animal experimental protocols were approved by the Animal Care and Use Committee of the Nanfang Hospital, Southern Medical University (NFYY-2017-42) and followed the National Guidelines for Animal Experimentation. All efforts were made to minimize the number of animals used and their suffering.

### Transient Focal Brain Ischemia Reperfusion Model

**FIGURE 1:**
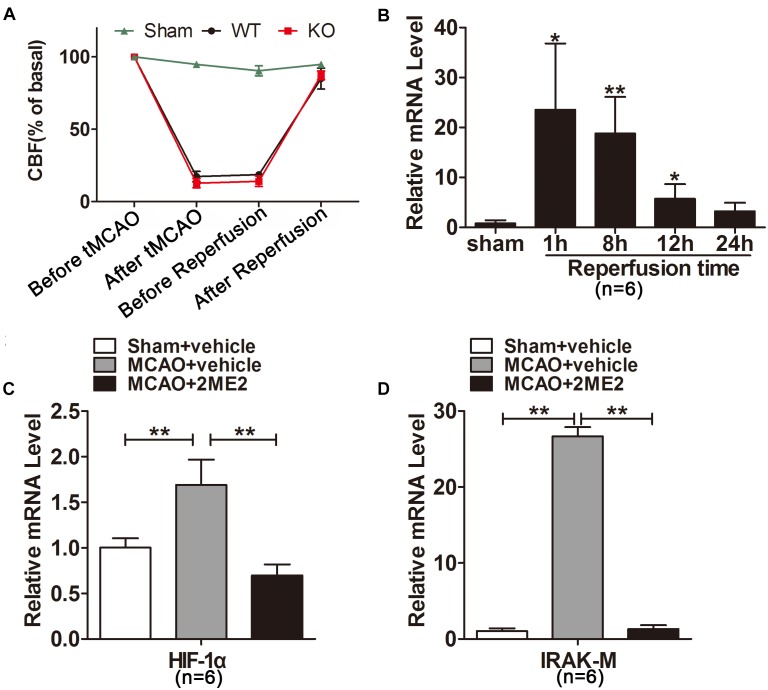
Interleukin-1 receptor-associated kinase (IRAK)-M was significantly increased after cerebral ischemia reperfusion. **(A)** Cerebral blood flow (CBF) of each mouse subjected to tMCAO detected by laser-Doppler and showed as percentage of basal level. **(B)** Relative mRNA levels of IRAK-M were measured by real-time RT-PCR at different time points after reperfusion in the infarcted cerebral hemisphere. Marked upregulation was noted after 1 and 8 h of reperfusion. **(C**,**D)** Effect of 2ME2 on the mRNA expression of HIF-1α **(C)** and IRAK-M **(D)** in the ipsilateral hemisphere at 1 h after reperfusion. Data are presented as mean ± SD, *n* = 6 per group. ^∗^*P* < 0.05, ^∗∗^*P* < 0.01 vs. sham group.

Mouse tMCAO was produced by filament occlusion of the right MCA to induce transient focal brain ischemia-reperfusion. In the pre-experiment, we found that 1 h of tMCAO caused a very high mortality in *Irak3* KO mice. Therefore, we chose 45 min tMCAO to conduct following experiments. In brief, mice were anesthetized with isoflurane (induction with 4%; maintenance with 1.5%) in oxygen-enriched air by face mask, and murine temperature was maintained at 37 ± 0.5°C throughout the experiment with heating pad. MCAO was performed by inserting a 7-0 nylon monofilament into the internal carotid artery via an external carotid artery stump and then by positioning the filament tip for occlusion at a distance of 8–9 mm beyond the internal carotid/pterygopalatine artery bifurcation. MCA was occluded for 45 min followed by reperfusion. Regional CBF (rCBF) was measured by the laser Doppler blood flow assessment (Moor Instruments, Wilmington, DE, United States) and presented as percentage of basal level (Figure 1A). The blunt needle probe of the laser Doppler blood flow assessment was positioned on skull surface in the MCA territory (1 mm posterior to the Bregma, and 5 mm lateral to the midline on the right parietal skull). Regional CBF was detected at four time points: before and after tMCAO; before and after reperfusion. Mice that did not show a >75% rCBF reduction or a <60% rCBF reperfusion over baseline levels or died after ischemia induction were excluded.

### Drug Administration

In order to study whether HIF-1α is associated with the expression of IRAK-M, WT mice were injected with 2-methoxyestradiol (2ME2) to suppress the expression of HIF-1α. Animals were randomly divided into different groups. The inhibitor of HIF-1α, 2-methoxyestradiol (2ME2, MedChemExpress, NJ, United States), was dissolved in dimethyl sulfoxide (DMSO) and further diluted in saline. 2ME2 solution was administered (5 mg/kg, i.p.) at 10 min before tMCAO. The WT mice in vehicle groups were intraperitoneally injected with same volume of DMSO diluted in saline.

### Neurological Tests

At 24 h after reperfusion, the global neurological and motor function of the animals were assessed using a modified Bederson Score (Table 1) and the grip test (string test) (Denorme et al., 2016) by a researcher blinded to group allocation. For the grip test, a length of string (50 cm) was pulled tight between two vertical supports and elevated 40 cm from a flat surface. The mouse was placed midway on the string and rated as shown in Table 1.

**Table 1:** Neurological function score.

Score	A modified Bederson score	Score	The grip test
0	No deficit.	0	Falls off.
1	Forelimb flexion.	1	Hangs onto string by one or both forepaws.
2	As for 1, plus decreased resistance to lateral push.	2	As for 1, and attempts to climb onto string.
3	Unidirectional circling.	3	Hangs onto string by one or both forepaws plus one or both hind paws.
4	Longitudinal spinning or seizure activity.	4	Hangs onto string by fore and hind paws plus tail wrapped around string.
5	No movement.	5	Escape (to the supports).

### Evaluation of Infarct Size and Hemispheric Brain Swelling

After 45 min of MCAO and 24 h of reperfusion, brain tissues at 1.0–8.0 mm posterior to the frontal pole were sliced coronally at a thickness of 1 mm and stained with 1% TTC (Sigma-Aldrich, St Louis, MO, United States). Infarct volume was then measured with using ImageJ software (NIH, Bethesda, MD, United States) and presented as percentage of the total brain volume by an experimenter blinded to group allocation (Wang et al., 2016). Hemispheric brain swelling was quantified according to the brain sections with the equation: hemispheric brain swelling = (volume of ipsilateral hemisphere/volume of contralateral hemisphere – 1) × 100%.

### Evaluation of BBB Permeability and Water Content

To evaluate the integrity of the BBB, EB leakage and IgG immunohistochemistry were performed at 24 h after reperfusion. EB was injected to mice via femoral vein (2% in normal saline at 4 ml/kg). At 24 h after reperfusion, animals were anesthetized and perfused with PBS. Then, cerebral hemispheres were incubated in 10 ml of methanamide (Macklin, Shanghai, China) at 60°C for 24 h and centrifuged. The supernatant was taken for spectrophotometric quantification of extravasated EB dye at by spectrophotometer (BMG Labtech, Offenburg, Germany).

For water content assay brain tissues were weighed on an electronic analytical balance to get the wet weight, and were dried in an oven at 60°C for 7 days to obtain the dry weight. Brain tissue water content was measured by the weighing the brain at wet and dry state and was calculated as (wet weight – dry weight)/wet weight × 100%.

### Western Blot Analysis

Brain tissues from the same region (2.0–4.0mm posterior to the frontal pole) were obtained with coronal cut, were divided into ipsilateral and contralateral hemispheres, and were homogenized in RIPA lysis buffer (Leagene, Beijing, China) containing protease and phosphatase inhibitors. After denatured in loading buffer, proteins were separated by SDS-PAGE (8–10% acrylamide) and then transferred to polyvinylidene difluoride membranes (Millipore, Billerica, MA, United States). After blocked by 5% non-fat milk, the membranes were incubated overnight at 4°C with primary antibodies including anti-COX-2 (1:1000; CST, Beverly, MA, United States, RRID:AB_2571729), anti-TNF-α (1:500; Santa Cruz; CA, United States, RRID:AB_1567355), anti-IL-10 (1:500; Santa Cruz, RRID:AB_10859554), anti-NLRP-3 (1:1000; NOVUS, CO, Cat# NBP2-12446), anti-iNOS (1:100; Santa Cruz, RRID:AB_627810), anti-p65 (1:1000; Proteintech, Chicago, IL, United States, RRID:AB_2178878), anti-β-actin (1:1000; Proteintech, Chicago, IL, United States, RRID:AB_2289225), and anti-TBP (1:500; Proteintech, RRID:AB_10951514) followed by incubation with anti-rabbit or anti-mouse horseradish peroxidase-conjugated secondary antibodies (1:5000; Santa Cruz). Bands were detected by chemiluminescence technology with using enhanced chemiluminescence advance Western blotting detection reagents (FDbio, Hangzhou, China), quantified and normalized to β-actin using ImageJ by an experimenter blinded to group allocation. The pictures of protein bands were opened by ImageJ and maximized to an appropriate zoom level. A frame was drawn around each band to make the band in the center of frame. The densities of protein blots were measured by program in ImageJ. The final relative quantification values are the ratios of net target bands to net loading controls.

### Histological Examination

Mice were perfused with saline and 4% paraformaldehyde at 24 h after reperfusion. Brains were post-fixed and subsequently immersed in 15% sucrose and 30% sucrose. Based on TTC stained and EB images, we selected the region at 4.0–5.0 mm posterior to the frontal pole. Coronal brain sections were obtained by slicing with Cryostat Microtome (Leica CM1800, Heidelberg, Germany). To detect the BBB permeability, immunohistochemistry was conducted with antibodies against mouse IgG (CoWin Biotech, Beijing, China) in accordance with the protocol suggested by the supplier (Lin et al., 2017). One picture of immunohistochemistry was opened by ImageJ. Region of interest (ROI) was selected by H-DAB in select model mode. Then the IgG relative intensity was measured. The antibody against Iba1 was used to detect microglia. Cryosections were permeabilized with Triton X-100, and were blocked with donkey serum. Sections were incubated with primary antibody against Iba1 (1:100; Abcam, Cambridge, United Kingdom, RRID:AB_1141557) overnight at 4°C and then with donkey anti-rabbit secondary antibody-conjugated to Alexa488 (1:500; Abcam) for 1 h at 37°C. Four serial sections of each brain sample were observed, three similar fields were selected in the brain region surrounded by three black boxes (Figure 8C) under an Olympus Fluorview laser scanning confocal microscope (Olympus, Tokyo, Japan). The number of Iba1-positive cells was counted by using ImageJ by an independent investigator who did not know the animal grouping (Li et al., 2018).

### Quantitative Real-Time PCR

Brain tissues of the same region (2.0–4.0mm posterior to the frontal pole) were obtained with coronal cut and were divided into ipsilateral and contralateral hemispheres. Total RNA of ipsilateral and contralateral hemispheres was isolated by using Trizol (Takara), and 1 μg of total RNA was reverse-transcribed to cDNA using PrimeScript^TM^ RT Master Mix Kit (Takara). RT-qPCR was performed with Roche LightCycler480 System (Roche, Basel, Switzerland) by using SYBR Green master mixes (Takara). Quantification was performed by the delta cycle time method, with mouse β-actin used for normalization. The mouse-specific primers (Sangon Biotech, Shanghai, China) are listed in Table 2.

**Table 2:** Primers used for real-time PCR.

Genes	Primer sequences
β-Actin	SENS: CGTTGACATCCGTAAAGACC
IRAK-M	REVS: AACAGTCCGCCTAGAAGCAC
	SENS: CAGGTGTCCTTCTCCACTGTTCTTG
	REVS: GTGACCTCAGACTGGCTGCA
MMP-2	SENS: TGGCACCACCGAGGACTATGAC
	REVS: ACACCACACCTTGCCATCGTTG
MMP-9	SENS: GGAGCACGGCAACGGAGAAG
	REVS: CCTGGTCATAGTTGGCTGTGGTG
IL-1β	SENS: TCGCAGCAGCACATCAACAAGAG
	REVS: TGCTCATGTCCTCATCCTGGAAGG
IL-6	SENS: ACTTCCATCCAGTTGCCTTCTTGG
	REVS: TTAAGCCTCCGACTTGTGAAGTGG
TNF-α	SENS: GCGACGTGGAACTGGCAGAAG
	REVS: GCCACAAGCAGGAATGAGAAGAGG
IL-10	SENS: CTGCTATGCTGCCTGCTCTTACTG
	REVS: ATGTGGCTCTGGCCGACTGG
COX-2	SENS: CTGGTGCCTGGTCTGATGATGTATG
	REVS: GGATGCTCCTGCTTGAGTATGTCG
iNOS	SENS: ATCTTGGAGCGAGTTGTGGATTGTC
	REVS:TCGTAATGTCCAGGAAGTAGGTGAGG

### Statistical Analysis

Statistical analyses were conducted by using SPSS 20.0 (IBM, Armonk, NY, United States) and GraphPad Prism 5.0 (GraphPad, La Jolla, CA, United States). During experiments and analysis, the investigators were blinded to genotype and experimental group. Data points outside the 95% confidence interval were excluded from the data analysis. Comparison between two groups was performed by using a two-tailed *t*-test. Differences within multiple groups were examined by two-way ANOVA followed by the Bonferroni *post hoc* correction. The incidence of hemorrhagic transformation in two groups was compared by using a Chi-square test. Data were expressed as mean ± SD. *P* < 0.05 was considered as statistically significant.

## Results

### Cerebral Ischemia-Reperfusion Time-Dependently Induced IRAK-M Expression in Mice

To explore the response of IRAK-M expression to cerebral ischemia-reperfusion, we firstly conducted mouse model of tMCAO with different reperfusion time followed by determination of IRAK-M mRNA expression with real-time fluorescence quantitative PCR. Regional CBF (rCBF) of each mouse was measured and shown in Figure 1A. After MCA was occluded, CBF decreased from 190.10 ± 17.02 to 28.03 ± 10.25 IU. When the nylon monofilament was removed, CBF returned to 163.70 ± 17.92 from 29.96 ± 8.33 IU. Results showed that IRAK-M mRNA was significantly upregulated in the infarcted brain tissue. Moreover, its expression peaked at 1h after reperfusion, 23.53 ± 13.27 folds higher than sham operative mice, and decreased to 18.79 ± 7.33, 5.70 ± 3.00 and 3.23 ± 1.70 folds at 8, 12, 24 h after reperfusion (Figure 1B), suggesting the time-dependent response of IRAK-M expression in ischemia-reperfused mice.

Hypoxia-inducible factor 1α is a key transcription factor induced by cerebral ischemia (Zhu et al., 2014) and is possibly involved in the IRAK-M expression (Shalova et al., 2015). To investigate whether HIF1α was responsible for inducing IRAK-M upregulation after reperfusion, we inhibited HIF1α mRNA expression before tMCAO onset. The results displayed that 2ME2 markedly suppressed the expression of HIF-1α and IRAK-M at 1 h after brain ischemia compared with the vehicle treated group (Figures 1C,D), indicating that the increase of HIF-1α correlates with the upregulation of IRAK-M after cerebrovascular obstruction and reperfusion.

### IRAK-M Deficiency Enlarged Infarct Volume and Deteriorated Motor Function After Cerebral Ischemia and Reperfusion in Mice

**FIGURE 2:**
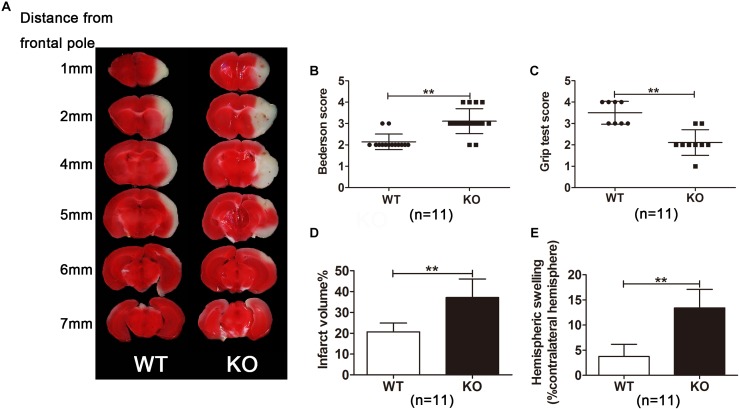
*Irak3*-null mice exhibited larger infarct volume and higher swelling index. **(A)** Representative images of TTC-stained brain slices from gene knockout and wild-type (WT) mice. **(B,C)** Neurologic deficit scores in WT and KO mice at 24 h after reperfusion. **(D,** **E)** Infarct size and swelling index were calculated with ImageJ software according to TTC staining. Data are presented as mean ± SD, *n* = 11 per group. ^∗^*P* < 0.05, ^∗∗^*P* < 0.01 vs. WT group.

Next, IRAK-M KO mice were used to observe the role IRAK-M in stroke mice. We assessed the neurological deficits and infarct volume of the mice via neurological function score and TTC staining, respectively, at 24 h after cerebral ischemia. Eleven mice were employed in both KO and WT groups to carefully observe the neurological outcome. Results showed that the deficiency of the IRAK-M led to a larger brain infarct volume (Figures 2A,D, *P* < 0.05 vs. WT) and deteriorated motor function deficit, including higher Bederson score and lower grip test score (Figures 2B,C, *P* < 0.05 vs. WT) compared with WT controls. We also observed hemispheric swelling on the basis of Figure 2A, and the data showed that the genetic deletion of IRAK-M significantly increased hemispheric swelling (Figure 2E, *P* < 0.05 vs. WT). These results demonstrate that IRAK-M deletion increases the infarct volume, and deteriorates the motor function of stroke mice.

### IRAK-M Deficiency Exacerbated Brain Edema and Hemorrhagic Transformation in Mice Model of Stroke

**FIGURE 3:**
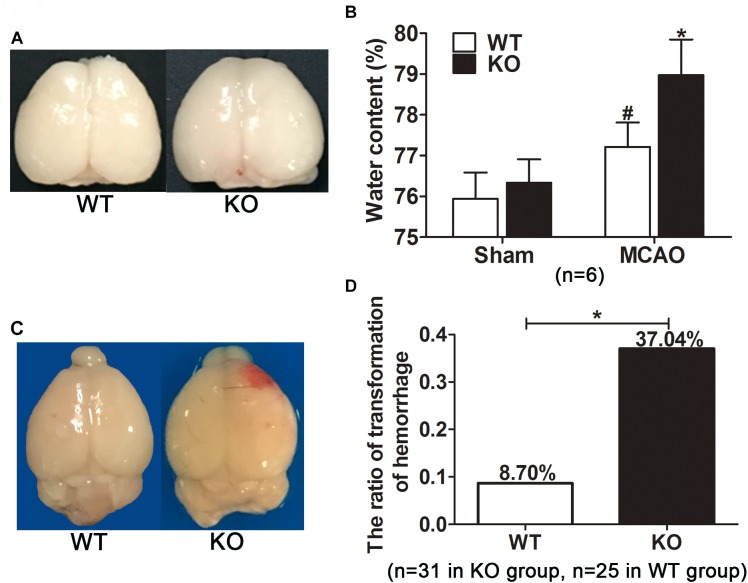
*Irak3* knockout increased brain water content and the incidence of cerebral hemorrhage transformation after reperfusion. **(A)** After 45 min tMCAO and 24h reperfusion, brains were isolated and photographs showing brain edema was captured. **(B)** Water content was measured by wet/dry method. Cerebral ischemia resulted in a marked increment in water content. IRAK-M deficiency accentuated brain swelling in MCAO mice compared with WT group. Data are expressed as mean ± SD, *n* = 6 per group. **(C)** Representative photographs showing the visible hemorrhagic foci appeared on the infarcted brain of the KO mice. **(D)** The incidence of cerebral hemorrhage transformation in each group was calculated. *n* = 31 in KO group and *n* = 25 in WT group. ^∗^*P* < 0.05 vs. WT group, ^#^*P* < 0.05 vs. sham group.

Based on the increased swelling index and the observed severe edema (Figure 3A) in KO mice, we further examined the effect of IRAK-M KO on water content in mouse brain subjected to tMCAO. Data revealed that, after 24 h of reperfusion, brain water content from ischemic KO mice was remarkably more than that in WT littermates (78.98 ± 0.87 vs. 77.22 ± 0.59, *P* < 0.05, Figures 3A,B).

During the process of brain isolation, the obvious hemorrhagic foci were found in KO mice (Figure 3C). Therefore, the incidence of visible hemorrhagic transformation was calculated among all included stroke mice. Twelve of the 31 KO mice developed visible hemorrhagic transformation after cerebral ischemia. In contrast, only two out of the 25 WT mice had intracerebral hemorrhage, much less than that in KO mice. In other word, IRAK-M deficiency remarkably increased the incidence of hemorrhagic transformation after cerebral ischemia-reperfusion (37.05% vs. 8.70%, *P* < 0.05, Figure 3D).

### IRAK-M Deficiency Increased BBB Permeability in Mice

**FIGURE 4:**
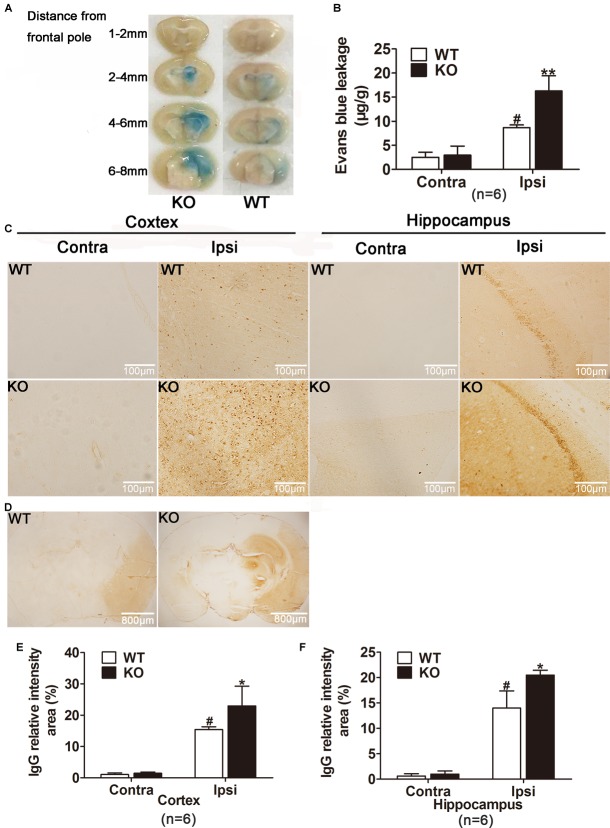
IRAK-M deficiency accentuated the extent of blood–brain barrier (BBB) leakage in mice. **(A,** **B)** Photographs and quantification of Evans blue extravasation are shown. Evans blue staining reveals an increase in the extent of BBB disruption in KO mice 24 h after reperfusion compared with that in WT mice. **(C)** Representative photomicrographs of IgG leakage in hippocampus and cortex at 24 h after reperfusion, respectively. Bar, 100 μm. **(D)** Representative microscopic images of brain sections after IgG staining. Bar, 800 μm. **(E)** The IgG relatively intensity was calculated with ImageJ and presented in the bar graphs. Data are expressed as mean ± SD, *n* = 6 per group. ^∗^*P* < 0.05, ^∗∗^*P* < 0.01 vs. WT group, ^#^*P* < 0.05 vs. sham group.

More remarkable brain edema and visible hemorrhagic foci in IRAK-M KO mice indicate more severe BBB leakage. Thus, temporal changes in BBB integrity were assessed by EB extravasation and IgG leakage after focal ischemic insult. As expected, EB leakage assay showed that the dye content in ipsilateral ischemic hemispheres was obviously higher in KO group than that in WT group (*P* < 0.05, Figures 4A,B), whereas no difference was found in contralateral hemispheres.

Moreover, endogenous IgG immunohistochemistry showing its extravasation in the brain was conducted as another approach to examine the location of BBB disruption. As shown in Figures 4C,D, ischemia reperfusion markedly increased IgG staining both in cortex and hippocampus of the ipsilateral hemispheres. Consistently, IRAK-M deficiency strikingly increased relative intensity of IgG staining in cortex and hippocampus (Figures 4C–F).

**FIGURE 5:**
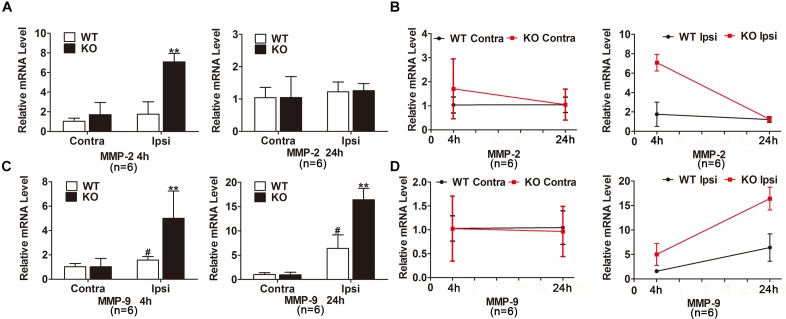
MMP-2 and MMP-9 expression were increased in the ischemic brain of KO mice. **(A**–**D)** Bar graphs and linear graphs showing the mRNA levels of MMP-2 **(A,B)** and MMP-9 **(C,D)** in ipsilateral and contralateral hemisphere of cerebral ischemia. The IgG relative intensity was measured using ImageJ. Data are expressed as mean ± SD, *n* = 6 mice per group. ^∗∗^*P* < 0.01 vs. WT group, ^#^*P* < 0.05 vs. sham group.

We next studied the potential mechanisms underlying the deteriorated BBB disruption after the deletion of IRAK-M. According to the Figures 5A–D, mRNA expression levels of MMP-2 and MMP-9 were robustly elevated in the injured brain tissue of KO group compared with WT group at 4 h after brain ischemia. After 24 h reperfusion, MMP-9, but not MMP-2, mRNA level increased in the ischemic cortex of KO mice compared with those of WT mice. Together, genetic deletion of IRAK-M accentuated BBB disruption via the upregulated expression of MMP-2 and MMP-9 in the mouse model of ischemic stroke.

### Reduction of IRAK-M Triggered Expression of Proinflammatory Factors in Mouse Brain After Cerebral Ischemia-Reperfusion

**FIGURE 6:**
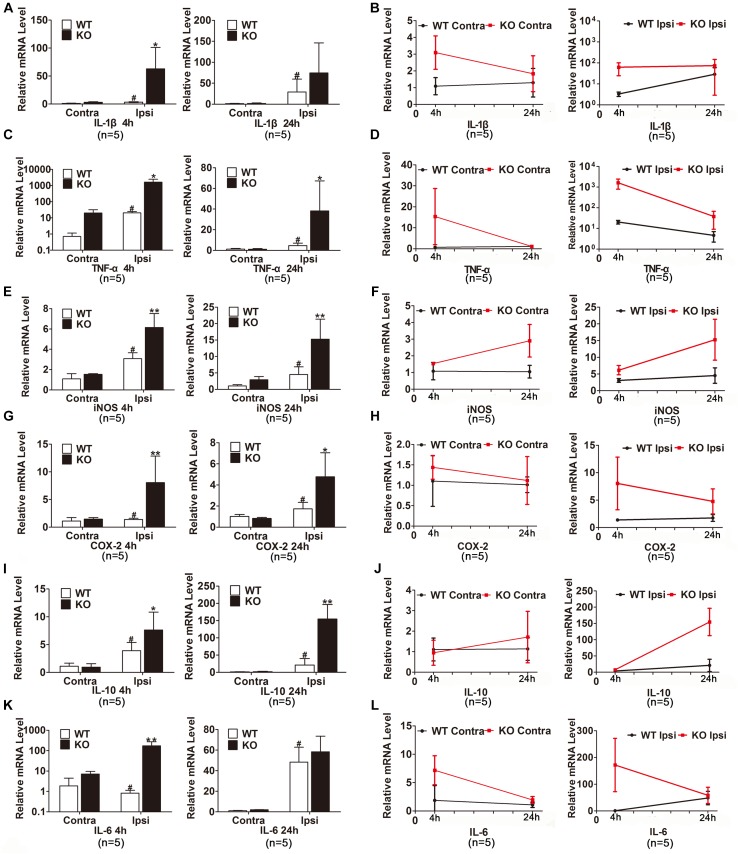
IRAK-M deletion further enhanced the mRNA levels of ischemia-induced inflammatory cytokines in mouse brains. **(A**–**L)** Bar charts and linear graphs showing the mRNA levels of IL-1β **(A,B)**, TNF-α **(C,D)**, iNOS **(E,F)**, COX-2 **(G,H),** IL-10 **(I,J),** and IL-6 **(K,L)** in brain tissues at 4 h and 24 h after reperfusion in WT and KO mice. *n* = 5 per group. ^∗^*P* < 0.05, ^∗∗^*P* < 0.01 vs. WT group, ^#^*P* < 0.05 vs. sham group.

**FIGURE 7:**
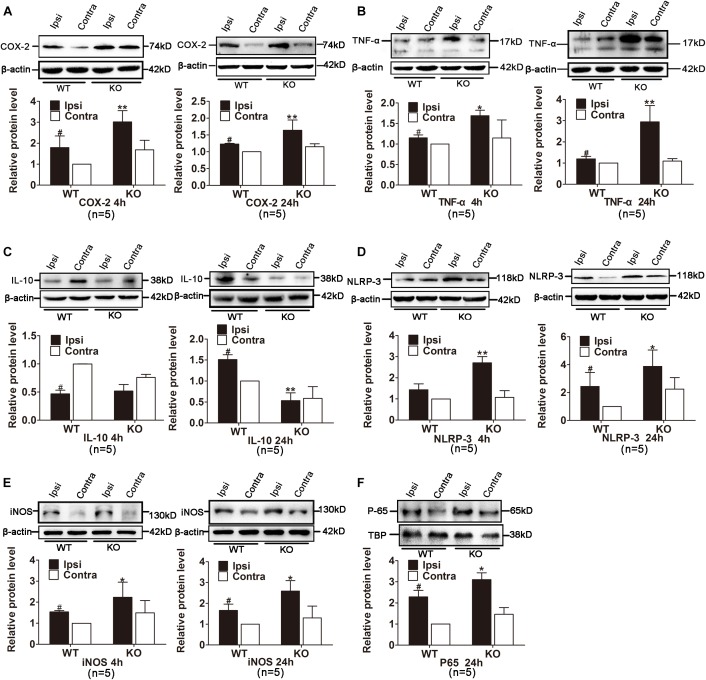
Effects of IRAK-M deletion on protein level of ischemia-induced inflammatory cytokines in mouse brains. **(A–E)** Representative Western blot images of COX-2 **(A)**, TNF-α **(B)**, IL-10 **(C)**, NLRP-3 **(D)** and iNOS **(E)** at 4 h and 24 h after stroke in KO and WT mice. β-Actin was used as the loading control. **(F)** Representative Western blot images of NF-κB P65 in nuclei in the ischemic hemisphere of KO and WT mice at 24 h after reperfusion. The relative protein level was quantified and normalized to β-actin using ImageJ. *n* = 5 per group. ^∗^*P* < 0.05, ^∗∗^*P* < 0.01 vs. WT group, ^#^*P* < 0.05 vs. sham group.

We also examined the expression of inflammatory cytokines in the ischemic hemisphere of the WT and IRAK-M KO mice. At the transcriptional level, the expression levels of IL-1β, TNF-α, iNOS, COX-2, IL-10, and IL-6 were dramatically elevated in IRAK-M KO mice at 4 and 24 h after reperfusion (Figures 6A–L). Moreover, the protein expressions of COX-2, TNF-α, NLRP-3, and iNOS were also upregulated at both time points (Figures 7A,B,D,E). However, the protein expression of IL-10, an anti-inflammatory mediator, was markedly decreased contrary to its mRNA expression (Figure 7C).

Those inflammatory mediators are either directly or indirectly triggered by NF-κB activation and inhibition of NF-κB activation is the critical step for the anti-inflammatory function of IRAK-M. We extracted proteins in nucleus to assess the localization of transcription factor P65 in the ischemic ipsilateral and contralateral hemisphere from different groups. Results in Figure 7F showed that the targeted deletion of IRAK-M induced more P65 nuclear translocation. In conclusion, these data suggest that, probably through inhibiting NF-κB signaling to alleviate inflammation, IRAK-M offers better neurovascular outcome in stroke mice.

### IRAK-M Deficiency Was Associated With Increased Microglia in tMCAO Mice

**FIGURE 8:**
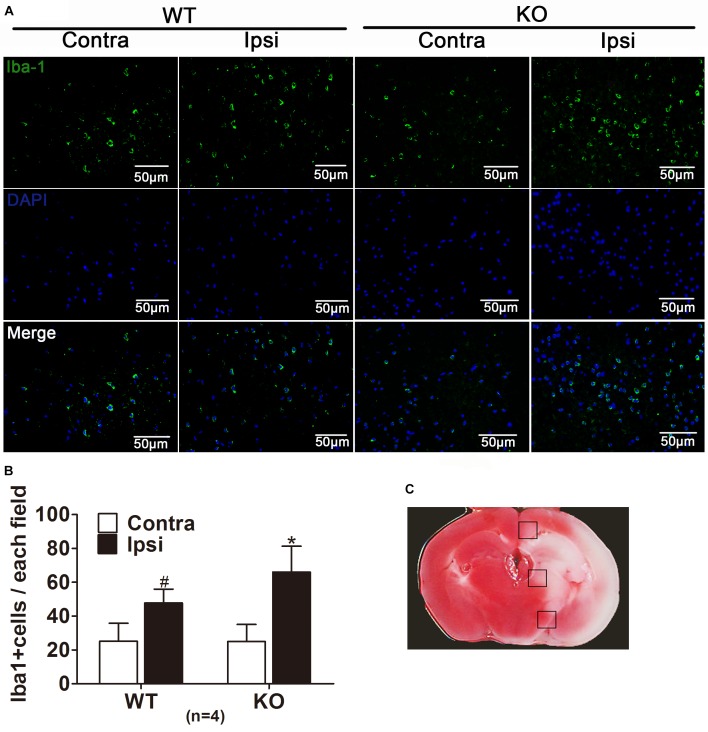
IRAK-M deficiency is associated with the enhanced microglia in tMCAO mice. **(A)** Representative pictures of immunofluorescence staining with anti-Iba1 antibodies (green) in the peri-infarct cortex at 24 h after reperfusion. **(B)** The number of Iba1-positive cells were counted and shown in bar graph, Scale bar = 50 μm. **(C)** TTC staining of ischemic brain manifested the location where the corresponding images were obtained. The brain region in black boxes indicates the areas where the typical Iba-1-positive cells were observed. The number of Iba1-positive cells was counted using ImageJ. *n* = 5 per group. ^∗^*P* < 0.05 vs. WT group, ^#^*P* < 0.05 vs. sham group.

Microglia is usually activated in the resident inflammatory environment and microglia leads to injury to BBB and exacerbates brain edema. To elucidate the effect of IRAK-M on microglia after stroke, we examined microglia activation in the cerebral cortex in the WT and *Irak3* KO mice at 24 h after reperfusion. As illustrated in Figure 8, the number of microglia, visualized by the staining of Iba1 antibody that specially binds to microglia common antigen, was significantly elevated at 24 h after reperfusion in KO mice compared with WT littermates (66.00 ± 15.28 vs. 47.80 ± 8.17), suggesting the increased microglia also participate in the exaggeration of neurovascular injury under the background of IRAK-M deficiency.

## Discussion

IRAK-M is an endogenous negative regulator of TLR signaling that prevents uncontrolled inflammation after tissue injury in multiple diseases. IRAK-M prevents dissociation of IRAK-1 and IRAK-4 from MyD88 and inhibits formation of IRAK-TRAF6 complexes and subsequent IκB degradation and NF-κB nuclear translocation. Nevertheless, its role in the cerebrovascular disorders is unclear. Here, we are the first to show that IRAK-M is increased in the infarcted brain, and its expression correlates with the increased HIF1-α expression. Deletion of *Irak3* aggravates functional deficits, cerebral infarct volume, brain edema, and causes hemorrhage transformation and BBB disruption. Meanwhile, we further demonstrate that IRAK-M deficiency offers increased expression of the mRNA and proteins of proinflammatory molecules, and enhances the activation of transcription factor NF-κB in ischemic brain regions. In summary, IRAK-M contributes to protect the brain from post-ischemic inflammatory response.

Ischemia-reperfusion that may contribute to the increased expression of IRAK-M has been, respectively, demonstrated in liver (Izuishi et al., 2006), heart (Chen et al., 2012), and brain (Wang et al., 2016). Compared with previous study at a single time point, the present study further revealed that the mRNA level of IRAK-M was dramatically upregulated at 1 h after cerebral ischemia reperfusion, and gradually downregulated in 24 h in the ischemic hemisphere, suggesting IRAK-M plays a role in acute stage of ischemic stroke. The effect of IRAK-M on infarct development hinges on their availability at early stage of ischemic stroke, consistent with the therapeutic window (<4.5 h).

The expression of HIF-1α is a key event in the pathophysiology of cerebrovascular diseases (Wu et al., 2013; Zhang et al., 2017). In the early phase of ischemic stroke, exposure of mice brain to the hypoxic stimulus results in an induction of HIF-1α protein expression throughout the brain (Zhu et al., 2014). More importantly, HIF-1α is a transcription factor that induces IRAK-M expression in monocytes in the mice model of sepsis (Shalova et al., 2015). In line with this finding, we found that the increased IRAK-M in cerebral ischemic hemisphere was almost completely reversed by 2ME2, further indicating HIF-1α is involved in IRAK-M mRNA expression in stroke mice.

Brain edema and hemorrhagic transformation are two leading causes for death of stroke patients (Bounds et al., 1981). Swelling of ischemic tissue causes enlargement of the infarcted area and early stroke deterioration of patients (Toni et al., 1995). Hemorrhagic transformation is another serious complication that restricts the application of thrombolytic therapy and brings secondary injury to brain tissues (Mao et al., 2017). In the present study, we observed that the deletion of the IRAK-M results in the poorer prognosis of stroke exhibiting much more severe brain edema and higher incidence of hemorrhagic transformation. These evidences suggest that IRAK-M is associated with the pathophysiology of brain edema and hemorrhagic transformation accompanying stroke. Moreover, quick early induction in response to brain ischemia-reperfusion may be in connection with alleviating the brain damages, and may provide additional ischemic tolerance against following dramatic injurious stimuli (Amantea et al., 2015; Wang et al., 2016). Some protective approaches, such as pre- or post- conditioning and neuroprotectants, may also induce IRAK-M production and offer neurovascular protection in stroke (Gidday, 2006; Izuishi et al., 2006). These interesting hypotheses deserve further investigation.

Both the brain edema and hemorrhagic transformation are linked to the disruption of BBB. Alleviation of the load of resident neuroinflammation has well been recognized to reduce BBB permeability (da Fonseca et al., 2014). When ischemia begins, the cytokines, chemokines, and immediate early genes initiate the release of extracellular MMPs which attack the basal lamina around the capillary, rupture endothelial tight junctional proteins and thus allow the extravasation of plasma proteins and opening of BBB (Nissinen and Kahari, 2014; Vafadari et al., 2016; Kempuraj et al., 2017). In addition, as early as 2 h after reperfusion began in rat brain, the BBB leakage was found in non-infarct area (Wang et al., 2016). Considering the anti-inflammatory role of IRAK-M and the early induction during cerebral ischemia-reperfusion, it is highly suggested that BBB is an ideal protective target for IRAK-M. Here, the targeted deletion of IRAK-M contributed to increased pro-inflammatory cytokines, enhanced MMPs expression, and more extravasation of EB dye and IgG in the ipsilateral brain. Moreover, brain-derived MMP-2 and MMP-9 activated by ischemia are associate with hemorrhagic transformation (Jickling et al., 2014), partly explaining the high incidence of hemorrhage of stroke KO mice.

The mechanism of the anti-inflammatory function of IRAK-M has been investigated by several groups. One study shows that, via stabilizing MKP-1, IRAK-M can suppress p38 activation (Su et al., 2007), while p38 activation can lead to MMP-9 increase in astrocytes (Gorina et al., 2011), composing a possibility that p38 activation in IRAK-M-null mice upregulates MMP-9 in the experimental stroke. We are conducting more experiments to testify this hypothesis. Meanwhile, IRAK-M prevented dissociation of IRAK1 and IRAK4 from Myddosome, and prevented formation of IRAK-TRAF6 complexes, subsequently inhibiting IκB degradation and NF-κB nuclear translocation (Akira and Takeda, 2004). Besides, IRAK-M is also able to mediate TLR7-induced MEKK3-dependent second wave NF-κB activation via interaction with IRAK-2 and inhibits the production of cytokines and chemokines at translational levels (Zhou et al., 2013). These are partly verified by our findings revealing NF-κB activation and the expression of target genes, including TNF-α, IL-1β, IL-6, iNOS, and COX-2, in KO stroke mice.

Although the expressions of pro-inflammatory factors are regulated at multiple levels, inhibiting NF-κB-mediated gene expression is believed to be a critical approach to restrict inflammation and protect neurovascular injuries in stroke (Lanzillotta et al., 2015). Among them, TNF-α, IL-1β and IL-6, iNOS and COX-2 are all dynamically altered in different stages of brain ischemia-reperfusion, and they usually bring detrimental effect to brain and BBB (de Vries et al., 1996; Lambertsen et al., 2012; Yagami et al., 2016). Considering the anti-inflammatory role of IRAK-M and the upregulation of IRAK-M in the first 24 h, we selected 4 and 24 h time points to test the expression of cytokines. Our study clearly revealed that KO mice exhibited extremely elevation of TNF-α, IL-1β, and IL-6, suggesting that the absence of IRAK-M can exacerbate the brain tissue injury of stroke through releasing larger amounts of proinflammatory cytokines in the acute phase of stroke.

Collectively, IRAK-M is promptly induced in response to cerebral ischemia-reperfusion. Genetic null mice displayed more severe neurologic deficits, brain edema, hemorrhage, and BBB disruption in stroke model. The exacerbation is related to the pronounced activation of NF-κB-mediated expressions of inflammatory factors in genetic null mice. Our study firstly provides evidence revealing that the function of IRAK-M in the brain and IRAK-M is potential to facilitate the development of new pharmaceuticals that reduce neurovascular complications. It is worth investigating the role of IRAK-M in other brain disorders and whether it functions as a regulatory target for neuroprotective therapy.

## Author Contributions

CL, YZ, MG, YH, and GL conducted the experiments. CW, ML, and SC performed statistical analysis. CL, YG, and SP wrote the manuscript. All authors read and approved the final manuscript.

## Conflict of Interest Statement

The authors declare that the research was conducted in the absence of any commercial or financial relationships that could be construed as a potential conflict of interest.

## Abbreviations

BBB: blood–brain barrier
CBF: cerebral blood flow
DAPI: 40,6-diamidino-2-phenylindole
EB: Evans blue
HIF: hypoxia-inducible factor
IKK: IκB kinase
IRAK-M: interleukin-1 receptor-associated kinase
KO: knockout
MAPK: mitogen-activated protein kinase
MCA: middle cerebral artery
MEKK: mitogen-activated protein kinase kinase
MMPs: metal metalloproteinases
MyD88: myeloid differentiation primary response 88
NF-κB: nuclear factor-κB
PRRs: pathogen recognition receptors
SPF: specific pathogen-free
tMCAO: transient middle cerebral artery occlusion
TLRs: toll-like receptors
TTC: 2,3,5-triphenyltetrazolium chloride
WT: wild-type
